# Wnt signalling in the development of axon, dendrites and synapses

**DOI:** 10.1098/rsob.180116

**Published:** 2018-10-03

**Authors:** Chun-Wei He, Chien-Po Liao, Chun-Liang Pan

**Affiliations:** Institute of Molecular Medicine, National Taiwan University College of Medicine, Taipei 10002, Taiwan, Republic of China

**Keywords:** Wnt, neuron, axon, dendrite, synapse

## Abstract

Wnts are a highly conserved family of secreted glycoproteins that play essential roles in the morphogenesis and body patterning during the development of metazoan species. In recent years, mounting evidence has revealed important functions of Wnt signalling in diverse aspects of neural development, including neuronal polarization, guidance and branching of the axon and dendrites, as well as synapse formation and its structural remodelling. In contrast to Wnt signalling in cell proliferation and differentiation, which mostly acts through β-catenin-dependent pathways, Wnts engage a diverse array of non-transcriptional cascades in neuronal development, such as the planar cell polarity, cytoskeletal or calcium signalling pathways. In this review, we summarize recent advances in the mechanisms of Wnt signalling in the development of axon, dendrite and synapse formation.

## 1. Introduction

Proper functioning of the nervous system depends on highly accurate and specific connectivity of neuronal circuits. In addition to genetic programmes governed by transcription factors that specify neuronal types and their wiring properties [1], extracellular signals are also instrumental in the construction of neural pathways. These secreted or membrane-tethered molecules provide a permissive environment that allows neurites to develop and extend. Moreover, some molecules directly instruct the trajectory of axon and dendrites [2]. Representative examples include neurotrophic factors, axon guidance cues and cell adhesion molecules [3]. Morphogens are secreted proteins that specify cell fate depending on their concentration gradients [4]. Classical morphogens, such as Hedgehog (Hh), transforming growth factor *β* (TGF-β) and fibroblast growth factors (FGFs), also play important roles in guiding migrating neurons or axon growth cones that are distinct from their canonical functions in controlling cell fate [5–7]. In this review, we focus on Wnts, an evolutionarily conserved family of morphogens that emerge as critical players in axon and dendrite development.

## 2. Wnt signalling

**Figure 1.**
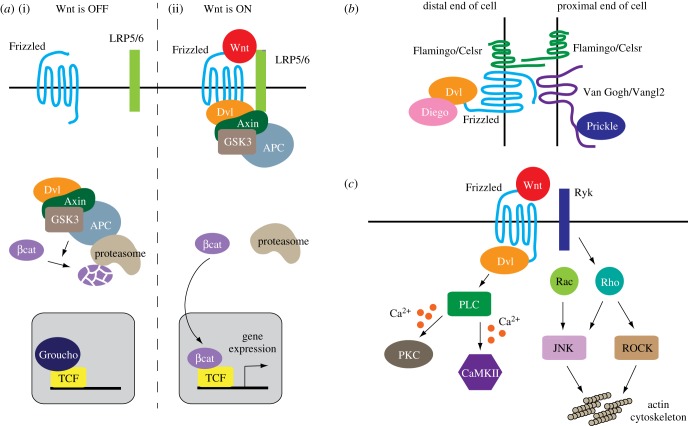
Wnt signalling pathways. (*a*) The β-catenin-dependent Wnt signalling. (i) In the absence of Wnts, the Axin-APC (Adenomatous polyposis coli)-GSK3β (glycogen synthase kinase 3β) complex promotes the proteasomal degradation of β-catenin. (ii) Activation of the Frizzled receptors by Wnts, in some cases with the help of the LRP5/6 co-receptor, relieves β-catenin from degradation. β-catenin is then translocated into the nucleus, displacing the transcriptional repressor Groucho to regulate gene transcription together with the TCF (T cell factor) transcription factor. Dvl, Dishevelled. (*b*) The Wnt-PCP pathway based on findings from the *Drosophila* epithelia. Frizzled, Dishevelled (Dvl) and Diego are localized to the distal end of the cell, where they interact with Van Gogh and Prickle at the proximal end of a neighbouring cell. Flamingo is distributed at both the proximal and distal ends of the epithelial cell. (*c*) The Wnt-calcium and Wnt-cytoskeleton pathways. Activation of phospholipase C (PLC) triggers calcium release from the endoplasmic reticulum, leading to the activation of calcium-sensitive effectors such as protein kinase C (PKC) and CaMKII. Wnt signalling can also activates the Rho and Rac small GTPases, leading to remodelling of the actin cytoskeleton via c-Jun N-terminal kinase (JNK) and Rho-dependent protein kinase (ROCK).

Present in metazoan species from cnidarians to primates, the Wnt family of secreted glycoproteins are well known for their diverse signalling functions [8,9]. Palmitoylated Wnts bind Frizzleds, which are seven transmembrane proteins that serve as cognate Wnt receptors, with the help of the LRP5/6 co-receptor in certain cases (figure 1*a*). The complexity of Wnt signalling comes in two flavours. First, the multiple homologues of Wnt ligands display relative, rather than absolute, binding specificity for the various Frizzled receptors, generating substantial promiscuity in ligand–receptor pairing [10,11]. Second, highly branched pathways relay signals downstream of Dishevelleds, cytoplasmic scaffolds that Frizzled receptors engage after Wnt binding (figure 1*b,c*). Major pathways downstream of Wnt-Frizzled signalling include one that regulates gene transcription through β-catenin (so-called canonical Wnt or Wnt-β-catenin pathway) and those that require polarity molecules, cytoskeletal elements or calcium signalling but are otherwise independent of β-catenin [8,9,12–14] (figure 1*b,c*). Adding to the complexity of Wnt signalling is its dependence on cellular and developmental contexts. As a classical morphogen, Wnts regulate cell fate largely through the β-catenin-dependent transcriptional pathway. Numerous studies suggest that β-catenin-independent cascades play crucial roles in controlling axon and dendrite development [15–17]. These Wnt signalling cascades will be examined in more detail in later sections that discuss individual aspects of axon or dendrite development.

## 3. Wnt signalling and axon development

### 3.1. Neuronal polarity

Neurons are highly polarized cells, as evidenced by the dendrites and axon being two morphologically and functionally distinct subcellular compartments. While the classic work by Dotti *et al.* [18] clearly established that axon–dendrite polarization can occur in dissociated hippocampal neurons, this finding does not exclude a role for extrinsic signals that may orient axon–dendrite polarity *in vivo*. The first evidence for Wnts as such *in vivo* polarity-orienting signals was provided by work with the nematode *Caenorhabditis elegans*. ALMs and PLMs are two pairs of mechanosensory neurons projecting a long anterior neurite and no (ALM) or a short (PLM) posterior neurite. The anterior dendrite of ALM and PLM has a single collateral branch that forms chemical synapses, while the posterior PLM neurite does not form synapses and has no known functions. The Wnt LIN-44 orients PLM polarity, while the other two Wnts CWN-1 and EGL-20 act jointly to polarize ALM [19–21]. In the absence of Wnts, the anterior neurite is truncated or absent, while the posterior neurite becomes long and forms synapses, causing a reversal of the apparent neuronal polarity [19–21], although a recent study argues that such morphological changes of neurons could be explained by Wnts acting as typical repulsive cues for the neurite [22]. In this regard, it is noteworthy that directional Wnt signals break the symmetry of *C. elegans* early embryonic cells and orient the mitotic spindle to enable subsequent asymmetric division of the blastomeres [23,24]. Directional Wnt signals also instruct the asymmetric division of blast cells in *C. elegans* larval ectodermal lineages [25–28]. A study with cultured embryonic stem cells shows that Wnts immobilized on beads and presented to the stem cells serve as local instructive signals to orient the plane of cell division, causing two daughter cells to be distinct in both size and gene expression patterns [29]. These studies indicate that for mitotic *C. elegans* blast cells, Wnts act as instructive signals that specify the direction of asymmetry in cell division. It remains to be tested whether Wnts directly specify the polarization of ALM, PLM and other postmitotic *C. elegans* neurons.

In *C. elegans*, genes in the planar cell polarity (PCP) pathway, one of the β-catenin-independent Wnt cascades, maintain the polarity of VC4 and VC5 motor neurons that control egg laying [30]. *dsh-1/Dishevelled*, *vang-1/Vangl2/Strabismus* and *prkl-1/Prickle* maintain VC4 and VC5 polarity with regard to the anterior–posterior body axis. In the *dsh-1*, *vang-1* or *prkl-1* mutants, VC4/5 motor neurons show incorrect polarization and frequently generate one supernumerary neurite [30]. While *prkl-1* acts in the VC4/5 neurons, *dsh-1* and *vang-*1 act in both VC4/5 and adjacent hypodermal cells to regulate VC4/5 neuronal polarity [30]. Mutations in *fmi-1/Flamingo*, another major PCP gene, also cause low-penetrance defects in the anterior–posterior polarization of *C. elegans* VD motor neurons, and *fmi-1* seems to act cell non-autonomously in VD polarization [31]. These findings echo the complex non-autonomous functions of PCP components in the classical *Drosophila* epithelial models of Wnt-PCP signalling [32,33].

In cultured rat hippocampal neurons, Wnt5a induces the activation of atypical protein kinase C (aPKC) via Dishevelled 1, which drives axon differentiation by functioning with PAR3 and PAR6, two proteins with known roles in cell polarization and axon specification [34,35]. Of note, glycogen synthase kinase 3*β* (GSK-3*β*), which is inhibited by Wnt signalling, needs to be inactivated in the nascent neurite that is specified to be the future axon [35]. These *in vitro* studies support a role of Wnts in instructing axon–dendrite polarization by engaging canonical polarity proteins. However, in *Drosophila*, aPKC, Par3 and Par6 are found to be dispensable for axon–dendrite specification [36]. Therefore, the role of molecules in neuronal polarity obtained from studies of dissociated neuronal cultures needs to be tested *in vivo*, as parallel or redundant pathways could exist and compensate for the loss of Wnt or other polarity signalling in axon–dendrite polarization.

### 3.2. Axon guidance

**Figure 2.**
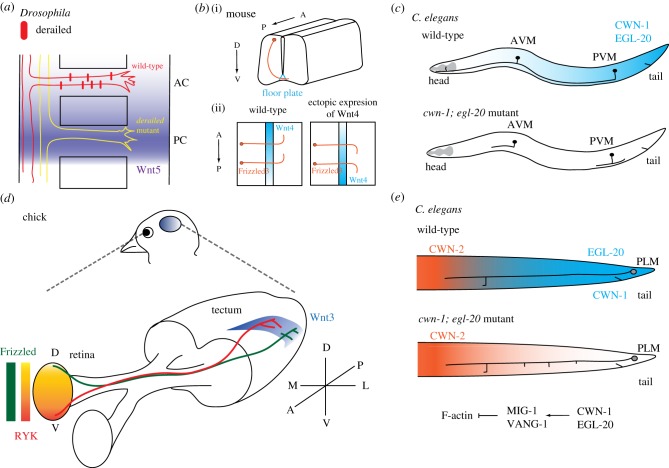
Wnt signalling and axon development. (*a*) Choice of the anterior versus posterior commissural routes in the *Drosophila* embryonic nervous system. Repulsive Wnt5 (purple) from the posterior commissure directs commissural axons to the anterior route via the Ryk/Derailed receptor. In the *Derailed* mutant, commissural axons enter the posterior route because of lack of repulsion. (*b*) Commissural axon guidance along the anterior–posterior (A–P) axis of the mouse spinal cord. (i) Oblique frontal view; (ii) view from the top of an open and flattened spinal cord preparation near the floor plate. The growth cones of the postcrossing commissural axons are attracted by Wnt4. (*c*) The A–P guidance of the AVM and PVM neuronal processes in *C. elegans*. CWN-1 and EGL-20 Wnt gradients high in the posterior repel the AVM and PVM processes to facilitate their anterior projection. In the *Wnt* mutants, the AVM and PVM terminate their anterior projection prematurely or develop ectopic projection to the posterior. (*d*) Topographic branching of the retinotectal axons in the chick optic tectum. Graded distribution of Wnts, indicated by blue colour shading along the medial-lateral axis of the optic tectum, instructs branching patterns of retinal ganglion cell (RGC) axons in their target zone. Ventral RGC axons branch away from the Wnt source as they express a high level of the repulsive Ryk receptor. Dorsal RGC axons that express the Frizzled3 receptor, by contrast, branch towards the Wnt source as Frizzled3 transduces attractive signalling at low Wnt concentration. The overall effect of Wnts is a biased branching of RGC axons laterally in the optic tectum. (*e*) Opposing Wnt gradients along the A–P axis (posterior EGL-20 and CWN-1, and anterior CWN-2) of *C. elegans* restrict the collateral branch of the PLM neuron to a narrow zone where the concentration of Wnts is at the trough. In this case, the Wnt-Frizzled signalling acts with VANG-1/Van Gogh to restrict F-actin assembly. Loss of the posterior Wnts leads to a shift of PLM branching positions towards the posterior.

Following the demonstration of Sonic Hedgehog (Shh), a morphogenic molecule, as an axon guidance cue for commissural axons in the mouse spinal cord, studies in both invertebrates and vertebrates revealed Wnts as instructive signals for axon pathfinding [37]. The evidence came when Yoshikawa *et al.* [38] first showed that midline-crossing axons in the *Drosophila* embryonic nerve cord project through the anterior commissure as a result of a repulsive Wnt5 signal from the posterior commissure (figure 2*a*). This study substantiated a prior work showing that overexpression of Wnt5 disrupted commissural axon guidance in *Drosophila* [39]. In another study, Lyuksyutova *et al.* [40] revealed that Wnt4 attract postcrossing commissural axons to project rostrally in the mouse developing spinal cord (figure 2*b*). Similar to signalling by other guidance factors, the type of receptor governs the signalling specificity of Wnts: the atypical receptor tyrosine kinase Derailed/Ryk mediates repulsive Wnt5 signalling in the *Drosophila* embryonic nervous system, and Frizzled3 transmits attractive *Wnt4* signalling in the mouse spinal cord [38,40]. Recent studies using regional and inducible Frizzled3 knockout mice show that Frizzled3 is required for the proper guidance of corticothalamic, corticospinal and thalamocortical axon tracts [41]. A study in *C. elegans* showed that Wnts repelled AVM and PVM mechanosensory neurites in the *C. elegans* ventral nerve cord [42] (figure 2*c*). Moreover, it demonstrated remarkable functional redundancy between different Wnt ligands or Frizzled receptors [42]. It should be noted that in *C. elegans*, Wnt-Frizzled signalling was previously shown to control the migration of neuroblasts [43–47], although in some of these cases, Wnts promote neuronal migration by initiating *Hox* genes expression and likely specifying transcriptional programmes that govern the behaviour of migrating neurons [43–45]. This mode of action is distinct from other paradigms where Wnts function as instructive signals and provide spatial information to guide migrating neurons and axon growth cones (see below). Subsequent studies have confirmed the importance of Wnt signalling in various axon guidance contexts, including the mouse corticospinal tract [48] (repulsive), serotonergic and dopaminergic brainstem axons [49] (both attractive and repulsive), retinal ganglion axons [50], axons of the medium spiny neurons in the striatum [51] and the corpus callosum [52,53], and *Drosophila* mushroom body axons [54,55].

Evidence that Wnt signals are instructive for growth cone migration came from both vertebrate and invertebrate studies. In open-book preparations of mouse spinal cord that make commissural axons accessible for manipulation, turning of postcrossing axons was found to be steered by attractive Wnt4 proteins locally released from Wnt4-expressing COS cells [40] (figure 2*b*). Site-specific expression of a repulsive EGL-20/Wnt protein in *C. elegans* also confirmed that EGL-20 repels neurite growth cones based on its concentration gradient [42].

Wnt signalling pathways downstream of the Frizzled or Ryk receptors that drive growth cone navigation are incompletely defined, but probably involve both β-catenin-dependent and independent pathways. In particular, components in the PCP pathway are implicated in various contexts of axon guidance or neuronal migration under Wnt signalling [15,49,55,56]. Canonical roles of the PCP proteins in polarizing epithelial tissues make them potential candidates to translate directional Wnt signals into the polarization of the motile axon growth cone, a function that would be difficult to envision with the β-catenin-dependent signalling that modulates gene transcription. The critical importance of Wnt-PCP signalling in growth cone guidance is well supported by many studies that reveal a requirement for various PCP proteins in axon development and navigation. *Celsr3*, a mammalian homologue of the PCP component Flamingo, which is an atypical cadherin with seven transmembrane domains, controls the development of several axon tracts in the mouse forebrain, including corticofugal and thalamocortical projections [57]. In spinal commissural axons, Vangl2, a four-pass transmembrane PCP protein homologous to *Drosophila* Strabismus/Van Gogh and *C. elegans* VANG-1, promotes Wnt signalling by facilitating internalization of Frizzled3 after activation by Wnt5 [56]. In this and a later study [58], data suggest that Vangl2 facilitates the endosomal localization of Frizzled3A in mammalian spinal cord neurons by antagonizing Dvl1/Dishevelled, which facilitates hyperphosphorylation of Frizzled3 and retains Frizzleds3 on the cell membrane. In a recent study, a similar role for VANG-1 to facilitate Frizzled internalization and the transduction of Wnt signalling is also found in *C. elegans* developing neurons, where VANG-1 colocalizes with Frizzleds and the two form protein complexes when expressed in cultured mammalian cells [59]. In the fly epithelia where many of the PCP components are first identified, Frizzleds and Van Gogh/Strabismus/Vangl2 are distributed to different ends of the epithelial cell. However, in either *C. elegans* or mammalian neurons, multiple lines of evidence suggest that they physically interact, form protein complexes or at least are in close proximity to each other [56,59,60].

Signalling downstream of the Ryk receptor in Wnt-mediated axon guidance is largely unclear. In the fly embryonic nervous system, repulsive Wnt5-Derailed/Ryk signalling requires members of the Src family kinases [61]. In the mammalian corpus callosum, axon guidance requires cytosolic calcium that rises after the activation of Ryk [53]. A recent study suggests that Ryk genetically interacts with PCP components in the cochlea [62], but whether the Wnt-Ryk signalling engages PCP components in other neurons or motile axons remains unexplored.

### 3.3. Axon branching

Collateral or terminal branching allows one single axon to innervate multiple target cells and is an essential mechanism that shapes the connectivity of neuronal circuits. Wnt7a signalling has been shown to promote terminal branching of cultured cerebellar granular neurons (GCs) [63]. As these neurons express Wnt7a, Wnts probably function as autocrine signals to promote neuronal branching. Terminal branching of the axon could also be regulated by target-derived Wnts. For example, in the mouse spinal cord, motor neurons of the lateral column secret Wnt3 to increase terminal arborization of the dorsal root ganglion (DRG) sensory axons that innervate them [64]. Branching of the sympathetic neuron presents yet another intriguing case, where autocrine Wnt5a from these neurons promotes their branching, yet the expression of Wnt5a is under the control of target-derived nerve growth factor (NGF) [65].

*In vivo* studies suggest that in addition to trophic support for axon branching, Wnt signals also instruct spatial patterns of axon branching. Projection of axons of the retinal ganglionic cells (RGCs) to their target neurons in the optic tectum, which is equivalent to the mammalian superior colliculus in amphibians and avian species, displays topographic organization [66,67] (figure 2*d*). RGC axons from the anterior or nasal retina project to the posterior tectum, and RGC axons from the posterior or temporal retina project to the anterior tectum. Several gradients of ephrins and Eph receptors in the optic tectum instruct RGC axon guidance [66,68,69]. In addition, ventral RGC axons project to the lateral tectum, and dorsal RGC axons project to the medial tectum (figure 2*d*). Schmitt *et al.* [69] found that a decreasing Wnt3 gradient from the medial to lateral tectum repels ventral RGC axons that express a high level of Ryk. Ectopic Wnt3 expression in the ventricular (lateral) zone of the chick optic tectum repels the terminal zone of the ventral RGC axons, confirming Wnts as a repulsive signal. Interestingly, dorsal RGC axons that express Frizzled3 are attracted by lower concentrations of Wnt3 [69]. This biphasic responsiveness to a single Wnt3 gradient enables RGC axons along the ventral–dorsal axis of the retina to project to the lateral–medial axis of the optic tectum.

A recent study in *C. elegans* also suggests that directional Wnt signals instruct where collateral neurite branches form by repulsion [59] (figure 2*e*). The *C. elegans* PLM mechanosensory neuron has a single collateral branch that comes off the major neurite trunk at stereotyped positions. In the *mig-1/Frizzled* or *vang-1* mutants, sites of the PLM branch become randomized, shifting to more proximal or distal positions. Genetic depletion of Wnts recapitulates this phenotype, while ectopic Wnt expression repels the branching sites away from the Wnt source [59]. In the wild-type, a single F-actin patch develops at a future PLM branching site. In the *cwn-1/Wnt*, *egl-20/*Wnt, *mig-1/Frizzled* and *vang-1* mutants, the abundance of F-actin is dramatically increased, together with widespread ectopic F-actin foci along the pre-branching nascent PLM process. Elimination of the Rac small GTPases MIG-2 and CED-10, which are critical F-actin assembly factors, restores normal F-actin distribution and PLM branching pattern [59]. These observations pinpoint F-actin as an effector of Wnt signalling in neurite branching.

## 4. Wnt signalling and dendrite development

### 4.1. Dendrite outgrowth and guidance

**Figure 3.**
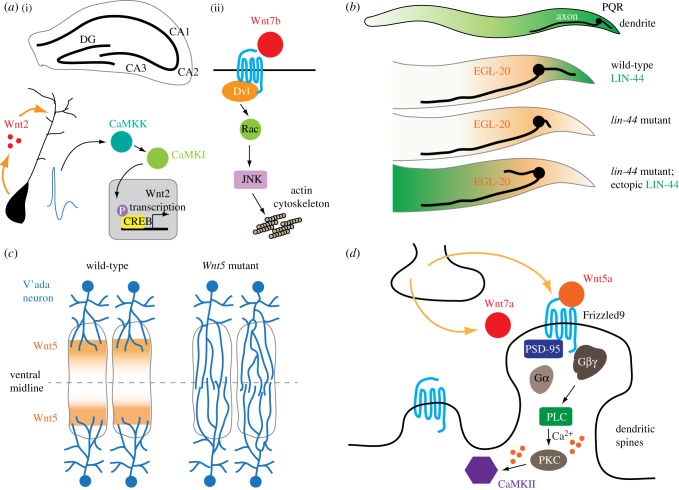
Wnt signalling and dendrite development. (*a*) (i) Activity-dependent Wnt2 transcription and secretion stimulate dendrite branching of the CA1 pyramidal neuron in the mouse hippocampus. (ii) Wnt7b also promotes dendrite branching through the Rac small GTPase and JNK. (*b*) A posterior LIN-44/Wnt gradient attracts the dendrite of the PQR neuron in *C. elegans*. Loss of LIN-44 results in defective posterior extension of the PQR dendrite, and an ectopic, reversed LIN-44 gradient can attract the PQR dendrite to the anterior. (*c*) In adult *Drosophila*, Wnt5 prevents the inadvertent invasion of the v'ada dendrites into the ventral midline or mixing with contralateral v'ada dendrites. (*d*) In the mouse, Wnts secreted from the presynaptic terminal enhances dendritic spine growth and maturation through trimeric G protein and calcium signalling, which culminates in the activation of CaMKII.

Similar to the axon, dendrite outgrowth and guidance are instructed by extrinsic signals. One example is the apical dendrite of the pyramidal neuron in the mammalian cortex, which is guided towards the pial surface by a gradient of attractive semaphorin 3A (Sema3A), a protein that repels axons [70]. Wnts have been implicated in activity-stimulated dendritic arborization [71]. In cultured rat hippocampal neurons, potassium-induced depolarization increases dendritic arborization that requires β-catenin and neuronal secretion of Wnts [71]. Interestingly, in this paradigm, β-catenin functions independently of the TCF transcription factor. Depolarization leads to the transcription of Wnt2 through calcium/calmodulin-dependent kinase I (CaMKI), CaMK kinase (CaMKK) and cyclic AMP response element-binding protein (CREB) [72] (figure 3*a*). In rat hippocampus, Wnt2 expression increases in the early postnatal period when the afferent projection is established in the dentate gyrus and CA1–3 regions [72]. Wnt7b stimulates dendrite development in dissociated mouse hippocampal neurons through Dvl1/Dishevelled, the Rac small GTPase and JNK, linking Wnt signals to the actin cytoskeleton, as Rac is a well-known actin regulator [73] (figure 3*a*).

The aforementioned studies of Wnt signalling in mammalian dendrite morphogenesis were mostly conducted using dissociated neuronal cultures or brain slices. A recent study on human patients of Williams syndrome, a neurodevelopmental disorder with hypersociability and mixed linguistic deficits, identified a mutation in the *frizzled9* (*FZD9*) gene as causative for the clinical disease [74]. Unexpectedly, neurons generated from patient-derived induced pluripotent stem cells (iPSCs) show longer total dendrite length and increased numbers of dendritic spines or synapses, which is confirmed in the analysis of a postmortem brain specimen [74]. Similarly, loss of the Ryk receptor results in excessive dendritic growth and branching in mouse hippocampal and cortical neurons [75]. Inhibitory effects of Ryk on dendrite growth are independent of its C-terminal PDZ-binding domain, suggesting that it may function independently of Dishevelleds or Vangl2 that bind this region [75].

An *in vivo* role for Wnts in dendrite outgrowth is revealed by observations made in *C. elegans*. Here, the projection of the dendrite of the *C. elegans* oxygen-sensing PQR neuron in the tail is instructed by attractive LIN-44/Wnt and EGL-20/Wnt signals that act through the LIN-17 and MIG-1 Frizzled receptors, respectively [76] (figure 3*b*). As is discussed in the section of *Neuronal Polarity*, defective dendrite guidance, rather than a reversal of neuronal polarization, has been proposed to explain the posteriorly directed mechanosensory dendrites in the *C. elegans* Wnt or Frizzled mutants [22]. In zebrafish, tiling of left and right trigeminal ganglionic axons along the dorsal midline of the head depends on mutual repulsion between axons, and ablation of unilateral trigeminal ganglion results in the invasion of contralateral trigeminal ganglionic axons [77]. In adult *Drosophila*, the v'ada sensory neurons from the left and right sides of the body tile their sensory territories along the ventral midline with the nonoverlapping distribution of their dendrites [78] (figure 3*c*). Interestingly, dendritic tiling of v'ada neurons does not depend on inter-neuronal repulsion; rather, repulsive Wnt5 signals from the ventral-most epidermal tissues keep dendrites away from the ventral midline [78] (figure 3*c*). Wnt5 acts through Ryk and Trio, a Rho GTPase exchange factor that has a well-established role in axon development [79,80]. This study highlights the similarity between axon and dendrites regarding Wnt-dependent guidance behaviours.

### 4.2. Dendritic spine formation

In vertebrate nervous systems, thorny protrusions called spines form on the dendrite shaft where postsynaptic densities are established. Dendritic spines are actin-rich dynamics structures that can grow or shrink in an experience-dependent way [81]; as such, they are often proposed to be a major anatomical substrate for neuroplasticity. In dissociated cultured neurons of the rat hippocampus, Wnt7a increases the density of dendritic spines and also promotes their transition into a mushroom-like morphology, which is an indicator of functionally competent excitatory synapses in the mammalian nervous system [82] (figure 3*d*). Wnt7a stimulates the activity of postsynaptic CaMKII, linking Wnt signalling to a well-established regulator of dendritic spine biogenesis [83]. In a recent study, Wnt5a is also shown to stimulate dendritic spine growth through Frizzled9 in the rat hippocampus [84] (figure 3*d*). This study further reveals an intriguing link of Wnt5a-Frizzled9 signalling to G*α*_o_ and G*β*γ-dependent increase of cytosolic calcium in the neuron [84]. As will be explored later, the role of Wnt5a in dendritic spine formation could also be related to its function in clustering PSD-95 (postsynaptic density protein 95), a scaffold protein critical for the assembly of postsynaptic structures [85].

A closer examination reveals an intriguing role of Wnt7a in dynamic spine strengthening associated with long-term potentiation (LTP), an electrophysiological enhancement of postsynaptic responses often implicated as part of the synaptic basis for learning [86,87]. It is shown that neuronal activation promotes Wnt secretion [71]. McLeod *et al.* [88] show that depolarization-induced Wnt7a secretion promotes dendritic spine growth, enhances postsynaptic response and localizes AMPA (α-amino-3-hydroxy-5-methyl-4-isoxazole propionic acid)-type glutamate receptors at dendritic spines. Wnt7a acts through Frizzled7 to activate protein kinase A (PKA) and CaMKII. PKA increases extrasynaptic AMPAR level, presumably expanding the size of the receptor pool that supplies AMPAR to the postsynaptic sites. By contrast, CaMKII inactivates SynGAP, a negative regulator of extracellular signal-regulated kinase (ERK) signalling that increases postsynaptic AMPAR level [88]. We would like to stress that in the mammalian nervous system, the dendritic spine is intimately associated with the synapses for its biogenesis and plasticity. As a result, it is sometimes challenging to delineate the causal relationship between spine biogenesis and synapse formation in some of the aforementioned studies on Wnt signalling. In the following section, we will focus on the role of Wnt pathways in synapse formation.

## 5. Wnt signalling and synapse development

### 5.1. Synapse formation and elimination

#### 5.1.1. Central synapses

**Figure 4.**
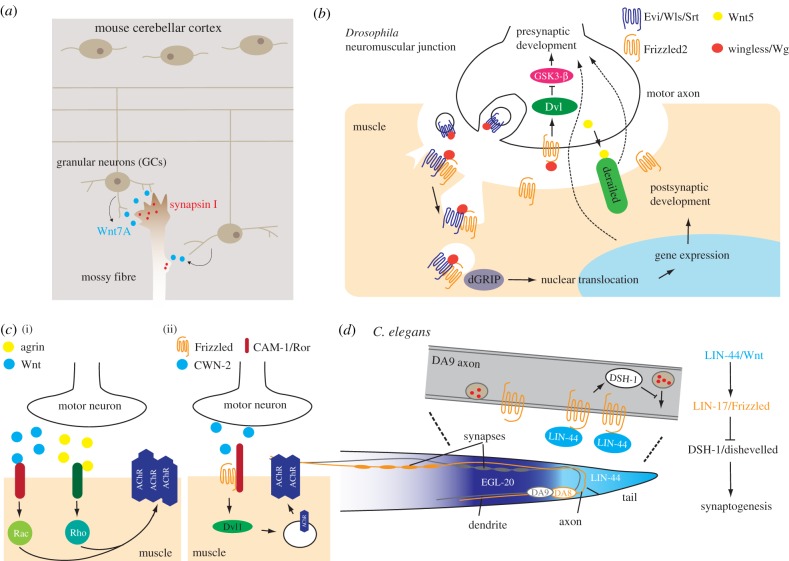
Wnt signalling in synapse development. (*a*) Wnt7a from the mouse cerebellar granular neurons (target) stimulates the expression and accumulation of presynaptic synapsin I in the mossy fibres (afferent axons). (*b*) At the *Drosophila* neuromuscular junction, Wnt-Frizzled signalling regulates the differentiation of the presynaptic and postsynaptic compartments in a distinct fashion. See the main text for details. Wls, Wntless. (*c*) In both mammals (i) and *C. elegans* (ii), Wnt-Frizzled signalling facilitates the clustering of the AChR at postsynaptic sites. AChR, acetylcholine receptor. (*d*) In *C. elegans*, repulsive Wnt signalling defines where *en passant* synapses form along the axon of the DA9 motor neuron. The Wnt signalling also ensures tiling of synapses between the DA9 and DA8 motor neurons, preventing inadvertent mixing of the presynaptic structures.

Lucas & Salinas [63] first observed that upon the addition of Wnt7a, the number of clusters of synapsin I, a presynaptic protein, was increased in cultured GCs. In this context, Wnt7a appears to act as an autocrine synaptogenic signal, as the GCs themselves secrete Wnt7a. GC-derived Wnt7a also acts upon the mossy fibres to increase the abundance of synapsin I and the complexity of cerebellar glomerular rosette, a multi-synaptic structure formed by the mossy fibre axon and multiple GC dendrites [89] (figure 4*a*). In *Wnt7a* null mice, synapsin I levels and ultrastructural complexity of the glomerular rosette are both markedly reduced in P8-9 littermates, suggesting that Wnt7a promotes synapse formation in the cerebellum. In the mouse hippocampus, Frizzled5 is located at both pre- and postsynaptic sites, where it promotes synaptogenesis after activated by Wnt7a [90]. The effect of Wnt7a on hippocampal synaptogenesis is neural activity-dependent and specific for excitatory synapses, and it also requires Dvl1/Dishevelled as well as Ca^2+^/Calmodulin-dependent protein kinase II (CaMKII) at the postsynaptic sites [82]. Wnt5a is also found to promote the differentiation of glutamatergic synapses in the rat hippocampus, evidenced by increased AMPA or NMDA (*N*-methyl-d-aspartate) neurotransmission [91].

One mechanism that Wnts use to stimulate the differentiation of synapses is by clustering or recycling of postsynaptic components. In dendritic spines of the rat hippocampus, Wnt5a promotes the clustering of PSD-95, a postsynaptic density scaffold protein of the membrane-associated guanylate kinase (MAGUK) family [85] (figure 3*d*). Intriguingly, Wnt5a also strengthens inhibitory synapses, facilitating membrane insertion and recycling of the postsynaptic GABA_A_ receptors via CaMKII [92]. Wnts can also promote synaptic differentiation through presynaptic mechanisms. As is discussed previously, Wnt7a increases the level of presynaptic synapsin I [63] and synaptic vesicle recycling [93] without altering postsynaptic structures (figure 4*a*). Wnts seem to function through distinct pathways to regulate different aspects of pre- or postsynaptic development [94]; in some cases, β-catenin is involved but seems to act independently of its function in gene transcription [93].

Studies exploring the Wnt-PCP pathways in synapse formation reveal somewhat contradictory conclusions on the role of Vangl2. In one such study, Vangl2 is shown to be required for dendritic spine formation and synaptogenesis by promoting N-cadherin endocytosis [95]. In heterozygous *Looptail* (*Lpt/+*) mice that carry a *Vangl2* mutation, dendritic spines are decreased in hippocampal CA1 pyramidal neurons [95]. By contrast, another study using conditional knockout mice induced to lose Vangl2 postnatally shows that the Vangl2-KO CA1 neurons have a more dendritic spine and increased frequency of miniature excitatory postsynaptic current (mEPSC) [96]. As PCP proteins are probably expressed in many types of neurons, cell non-autonomous influence of PCP components may confound these studies as they either use an *in vitro* system or pan-neuronal PCP-KO mice. Future studies that employ *in vivo*, cell-type-specific manipulation of Wnt-PCP pathways are needed to solve these discrepancies. Mutations in *fmi-1/Flamingo* disrupt the presynaptic organization in *C. elegans* DD and VD GABAergic motor neurons, manifesting as abnormal active zones, inappropriate apposition of presynaptic and postsynaptic structures, and extrasynaptic accumulation of synaptic vesicles [31]. *fmi*-1 is not expressed in VD neurons, suggesting that it acts cell non-autonomously probably through *cdh-4*, a cadherin homologue expressed in VD [31]. In *Celsr3/Flamingo* conditional KO mice, there is a dramatic reduction in hippocampal CA1 glutamatergic synapses [96]. It remains to be determined which Wnt ligands function as upstream signals for these synaptogenic PCP components.

#### 5.1.2. Peripheral synapses

In addition to central synapses, Wnts also regulate the formation of synapses in the peripheral nervous system. At *Drosophila* neuromuscular junctions (NMJs), Wingless/Wnt derived from the presynaptic terminal is required for the full spectrum of development in both pre- and postsynaptic compartments [97]. A surprising discovery of Wnt signalling at the fly NMJ is that binding of Wingless results in the nuclear translocation of the *Drosophila* Frizzled2 cytosolic terminus (DFrizzled2-C) [98,99] (figure 4*b*). Somewhat unexpectedly, the proteolytic cleavage and extranuclear transport of DFrizzled2-C does not require Wingless [98]. Presynaptic secretion of Wingless requires exosomes containing Wntless/Evenness interrupted (Evi)/Sprinter/MIG-14, a conserved regulator of Wnt intracellular trafficking and secretion [21,100–107] (figure 4*b*). Activity-dependent Wingless signalling also strengthens presynaptic functions via the inhibition of GSK-3β [99]. Thus, Wingless signalling bi-directionally regulates the *Drosophila* NMJ in an activity-dependent manner. A recent finding suggests that glial cells could be a source of synaptogenic Wingless signals in fly NMJs, and glia-derived Wingless clusters of glutamate receptors at the postsynaptic membrane [108].

Motor neuron-derived Wnt3 facilitates the aggregation of acetylcholine receptors (AChRs) at chick and mouse NMJs [109], which requires Dvl1 and the Rac1 small GTPase but not other components in the canonical Wnt pathways (figure 4*c*). Of note, AChR aggregation by Wnt3 is only evident when agrin, an AChR-clustering factor secreted from motor axon terminals, is present [109] (figure 4*c*). Wnt4 and Wnt11 are also shown to be important for NMJ development in mice, and they signal through both β-catenin and Vangl2 to promote AChR clustering [110]. In *C. elegans*, neuronally derived CWN-2/Wnt facilitates the translocation of ACR-16/AChR to the postsynaptic sites in the muscle via LIN-17/Frizzled, DSH-1/Dishevelled and CAM-1/muscle-specific kinase (MuSK), an atypical Wnt receptor [111] (figure 4*c*). At zebrafish NMJs, Wnt11r clusters AChR through postsynaptic MuSK [112]. Interestingly, retrograde signalling by muscle β-catenin also promotes the differentiation of presynaptic motor terminal [113]. There are also reports suggesting an inhibitory role of Wnt3 signalling in AChR clustering that requires β-catenin but not gene transcription [114]. As will be described below, Wnts inhibit synapse formation in some *C. elegans* motor neurons, a function that is opposite to what is found in the vertebrates and *Drosophila*. This observation highlights the diversity and complexity of Wnt signalling in the nervous system.

### 5.2. Spatial arrangement of synapses

The spatial distribution of synapses defines parts of neural circuit connectivity and is thus tightly regulated. In *C. elegans*, *en passant* synaptic regions between two adjacent longitudinal motor axons that target different muscles tile each other, forming mutually exclusive topographic distribution. Klassen & Shen [115] first showed that directional LIN-44/Wnt signals inhibit the formation of presynaptic structures, generating a synapse-free domain in the proximal region of the DA9 motor axon (figure 4*d*). Loss of LIN-44 or its cognate Frizzled receptor LIN-17 leads to the invasion of synapses into the proximal DA9 axon domain, whereas increased LIN-44 expression expands the synapse-free region on the axon. Since the polarity or axon trajectory of the DA9 neuron is not affected in the *lin-44* or *lin-17* mutants, these observations implicate LIN-44/Wnt as a repulsive signal for presynaptic structures. Mizumoto & Shen [116] further demonstrate that combinatorial Wnt gradients of LIN-44 and EGL-20 establish tiling of presynaptic structures between DA9 and the adjacent DA8 motor axon. As F-actin is a critical element in the assembly of presynaptic structures [117,118], and Wnts restrict F-actin distribution in *C. elegans* neurons [59], it is tempting to speculate that Wnts prevent synapse formation by locally inhibiting F-actin assembly.

## 6. Wnt signalling in axon/dendrite remodelling and maintenance

The architecture of a neuron is not static after its establishment. Rather, various scales of structural refinement and remodelling allow the neuron to shape its morphology in response to sensory experience or physiological signals. On the other hand, there is evidence that the neuronal architecture needs to be actively maintained for its structural and functional stability. Wnt5a begins to express in postnatal mouse hippocampus and expression continues throughout adulthood [119]. Wnt5a is important for maintaining dendritic morphology of the CA1 pyramidal neurons in adults, as in the *Wn5a* knockout mice, these neurons show normal morphology at birth but undergo a progressive loss of dendrite branches starting 4.5 months postnatally [119]. This structural deterioration is accompanied by impaired LTP induced by high-frequency stimulation and memory functions [119]. Wnt5a is secreted by CA1 neurons, suggesting that it acts as an autocrine, trophic factor to maintain dendrite architecture in adulthood. Importantly, hippocampal expression of exogenous Wnt5a starting at six months postnatally completely rescues the late-life defects in CA1 dendrite morphology, confirming an adult-specific role of Wnt5a in dendrite maintenance [119]. Interestingly, it has been reported that the expression of some Wnts and the Ryk receptor is increased after conditioning nerve crush injuries in adult rats [120,121]. Local introduction of Wnt4 causes retraction of the central axon of DRG neurons that have experienced a peripheral, conditioning injury. By contrast, secreted Wnt inhibitors, such as secreted Frizzled-related protein 2 (SFRP2) or Wnt inhibitory factor 1 (WIF1), enhance regeneration of the central axon [121]. These studies resonate with a long-held view that developmental signals that enter dormancy in the adult nervous system could be reactivated upon neural injuries. In a follow-up study, the same group reported that the Ryk-KO mice subjected to partial spinal cord injuries show increased axon collateral branching in the motor cortex, accompanied by an enhanced reorganization of motor cortex and recovery in forelimb functions [121]. Of note, the benefit conferred by *Ryk* deletion does not occur in the absence of rehabilitative training, suggesting that removal of a Wnt receptor improves neural circuit remodelling in an activity-dependent manner [121]. These studies represent some of the emerging efforts that address developmental signals as potential therapeutic targets for correcting neuronal defects occurring in adult, and they open exciting new avenues in the field of Wnt signalling research.

## 7. Conclusion

The past two decades have witnessed an exciting advancement in the mechanistic understanding of Wnt pathways in neural development. Given the general importance of Wnt signalling in the wiring of neuronal circuitries, it is important to acknowledge several remarkable challenges that remain after numerous studies. First, the signalling cascades downstream of the Frizzled or Ryk receptors remain incompletely defined in individual contexts of neuronal development. Second, the responsiveness to Wnts critically depends on the intrinsic property and the developmental stage of a given neuron, and little is known about the intrinsic programming as well as the dynamic nature of neuronal responsiveness to Wnts. Third, multiple homologues exist for Wnt ligands and Frizzled receptors. It is thus conceivable that a combinatorial ‘Wnt-Frizzled code’ operates to define axon or dendrite development, in particular, axon guidance and topographic patterns of neurite branching or synapse formation that rely on directional, instructive Wnt cues. Fourth, the cell non-autonomous nature of the function of several of Wnt signalling components, such as Frizzleds and PCP proteins, is likely to influence the interpretation of some of the neuronal phenotypes in respective mutants. *In vivo* models using sophisticated genetic and mosaic analysis are essential to solve the complex issues of non-autonomous Wnt-PCP signalling in neuronal development and function, such as the somatic CRISPR (Clustered Regularly Interspaced Short Palindromic Repeats) technique recently developed in *C. elegans* [122]. Moreover, emerging evidence indicates that Wnt signalling is involved in the maintenance and plasticity of the adult nervous system, a role that has received much less attention in the past compared with that of the Wnt pathways in neural development. Future progress in this direction could greatly advance our understanding of Wnt signalling in adult neuroscience, in particular, of fields related to neuroregeneration and neurorehabilitation [17]. Successful tackling of these challenges will hopefully produce a clearer picture of the dazzlingly complex Wnt signalling in the construction and function of the nervous system.

## Data accessibility

This article has no additional data.
